# Magnetic and elemental characterization of the particulate matter deposited on leaves of urban trees in Santiago, Chile

**DOI:** 10.1007/s10653-022-01367-w

**Published:** 2022-09-06

**Authors:** M. Préndez, C. Carvallo, N. Godoy, C. Egas, B. O. Aguilar Reyes, G. Calzolai, R. Fuentealba, F. Lucarelli, S. Nava

**Affiliations:** 1 Facultad de Ciencias Químicas y Farmacéuticas, Universidad de Chile, Sergio Livingtone 1007, Independencia, Santiago, Chile; 2 UMR 7590, Institut de Minéralogie, de Physique des Matériaux et de Cosmochimie, Sorbonne Université, 4 Place Jussieu, 75005 Paris, France; 3 Instituto Ciencias Biológicas, Universidad de Talca, Av Lircay s/n, Talca, Chile; 4 Unidad Morelia, Instituto de Investigaciones en Materiales, Universidad Nacional Autónoma de México, Antigua carretera a Pátzcuaro No 8701, Col. Ex Hacienda de San José de la Huerta, 58190 Morelia, Michoacán Mexico; 5 Department of Physics and Astronomy, University of Florence and National Institute of Nuclear Physics (INFN), Florence, Italy

**Keywords:** Urban air pollution, Vehicular emissions, Magnetic characterization, PIXE elemental quantification, Urban vegetation, Phytoremediation

## Abstract

Airborne particulate matter is a serious threat to human health, especially in fast-growing cities. In this study, we carried out a magnetic and elemental study on tree leaves used as passive captors and urban dust from various sites in the city of Santiago, Chile, to assess the reliability of magnetic and elemental measurements to characterize particulate matter pollution from vehicular origin. We found that the magnetic susceptibility and saturation isothermal remanent magnetization measured on urban tree leaves is a good proxy for tracing anthropogenic metallic particles and allow controlling the exposure time for particulate matter collection, in agreement with other studies carried out in large cities. Similar measurements on urban soil can be influenced by particles of detritic (natural) origin, and therefore, magnetic measurements on tree leaves can help to identify hotspots where fine particles are more abundant. Elemental particle-induced X-ray emission analysis of tree leaves showed the presence of a number of elements associated with vehicular emissions, in particular Cu, Zn, Fe, K and S which are present at every site, and As, Se, V, Ni, Sr, Zr, Mo and Pb identified at some sites. We observed a correlation between magnetic parameters and the concentrations of S and Br as well as Cu to a smaller extent. Moreover, this study shows the importance of selecting carefully the tree species as well as the location of trees in order to optimize phytoremediation.

**Supplementary Information:**

The online version contains supplementary material available at 10.1007/s10653-022-01367-w.

## Introduction

The increase in the urban population leads to the loss of natural landscape and its replacement by an artificially built environment; it is a global phenomenon, greater in Latin America. Consequently, urban air quality has been deteriorating and many cities currently experience pollution levels above the World Health Organization (WHO) recommended limits. Among others, particulate matter (PM) is a frequently found contaminant in cities. It is present in the atmosphere with different mass concentrations, sizes, shapes and chemical composition, because it comes from many different sources, atmospheric conditions and urban geometry in relatively small areas (Buccolieri et al., 2010; Hofman et al., 2013). Both acute and chronic exposure to inhalable particles PM with aerodynamic diameter ≤ 10 µm (PM_10_) is associated with various pulmonary, cardiovascular and neurological problems (Coccini et al., 2017; Costa et al., 2014; Liu et al., 2018; Malik et al., 2019; Pope & Dockery, 2006; Weichenthal et al., 2017).

In cities such as Santiago, the main sources of anthropogenic PM are vehicle usage, industrial activities and emissions from burning fossil fuel and wood. In many countries, both the mass concentrations of PM_10_ and PM_2.5_ (fine inhalable particles with aerodynamic diameter ≤ 2.5 µm) are included in the standardized outdoor air quality measurement. In Santiago, this monitoring is carried out with 11 stations managed by the Ministry of the Environment’s air quality monitoring network (SINCA: Sistema de Información Nacional de Calidad del Aire). However, many urban areas lack coverage and the network cannot characterize the temporal and spatial variability of PM concentrations (Karner et al., 2010; Zikova et al., 2017), hindering the estimation of exposure to PM and its effects in health, or the understanding of the effectiveness of emission reduction policies (Kelly et al., 2017; Tsai et al., 2019). Moreover, the ultrafine particles (UFP: fine inhalable particles with aerodynamic diameter ≤ 0.1 µm) are not considered in this monitoring. In Chile, particularly Santiago, concentrations of PM are especially high during critical episodes of pollution occurring in autumn–winter seasons (Préndez et al., 2011; Toro et al., 2014).

However, there is growing evidence that the removal of PM is one of the positive effects of urban forests (Dzierzanowski et al., 2011; Manes et al., 2016; Nowak et al., 2018; Song et al., 2015), among other effects such as providing primary productivity of vegetation (Costanza et al., 2007; Roeland et al., 2019) or removing gases (Araya et al., 2019; Nowak et al., 2018; Viecco et al. 2018). Indeed, the leaves’ surface and stems adsorb or absorb significant amounts of air pollutants. Although a fraction of this PM is resuspended by wind, another part remains attached to the plant. Moreover, particles with aerodynamic size less than 0.2 µm can be captured by the leaves through the stomata (Ottelé et al., 2010; Song et al., 2015; Egas et al. 2020). The effectiveness of this effect is species-specific. Dzierzanowski et al. (2011) showed that four species of roadside trees were able to purify urban air by dry deposition of PM on the leaves.

However, urban trees in Santiago are mainly exotic species (~ 86%), which lose their leaves in the autumn–winter period (Hernández & Villaseñor, 2018) of highest PM concentrations and therefore have little contribution to the PM decontamination process when it is most needed.

The quantification of PM in urban environment can be achieved using magnetic measurements (e.g., Matzka & Maher, 1999). Indeed, magnetic particles are present in PM from vehicular sources, because particles emitted as residuals from fuel burning and wear and friction of engine components are Fe-rich. These particles can also lodge in their structure other toxic metals such as Pb, Zn, Ba, Cd and Cr because of the affinity of Fe oxides with trace metals (Aguilar Reyes et al., 2011, 2012; Cao et al., 2015). Several studies have reported a good correlation between magnetic susceptibility and the heavy metal content (Mitchell & Maher, 2009; Muñoz et al., 2017; Wang, 2013; Yang et al., 2017). As a consequence, the magnetic method has been developed to provide a fast and inexpensive alternative for monitoring metallic fine particles in anthropogenic pollution, especially those coming from transport. The use of biological passive captors (tree leaves, bark, lichens, and moss) has been popularized because they are widely available in cities, sampled at breathing height, and provide a record of location-specific and time-integrated information on local air quality. A number of magnetic biomonitoring studies have been carried out recently in Latin American cities using such captors (e.g., Castaneda-Miranda et al., 2021; Chaparro et al., 2013; Marié et al., 2016).

The principal source of pollution by PM in Santiago is vehicular pollution (Gramsch et al., 2014). Moreover, other studies have shown that roadside tree leaves are mainly sensitive to the effect of vehicular pollution (e.g., Matzka & Maher, 1999; Moreno et al., 2003; Muñoz et al., 2017). Our study was divided into three subparts with different goals:

- To study spatial variation of PM along a section of a busy avenue
- To evaluate the ability of several tree species present in Santiago in trapping PM
- To assess the relevance of Air Official Monitoring Stations (AOMS) location by comparing the measurements close to the AOMS with measurements from the other two subparts.

These goals are addressed by means of standard magnetic measurement and in some cases elemental measurements, on tree leaves. Urban dust was also studied in some cases in combination with the tree leaves.

## Materials and methods

### Sampling

Santiago City is located in the Metropolitan Region and counts more than 7 million inhabitants (Instituto Nacional de Estadistica: https://www.ine.cl/estadisticas/sociales/censos-de-poblacion-y-vivienda/censo-de-poblacion-y-vivienda [Assessed 07/29/2022]). The chosen sampling sites are in the area (2 km of radius) covered by the air official monitoring stations (AOMS) installed in 11 Santiago communes.

At each site, 10–15 mature and well-exposed leaves were collected per tree at a height of about 1.75 m. When available, urban dust (UD) was swept from the asphalt close to the sampled trees (see Supplementary Information (SI) section for more details). The sampling was conducted over three campaigns.

**Fig. 1:**
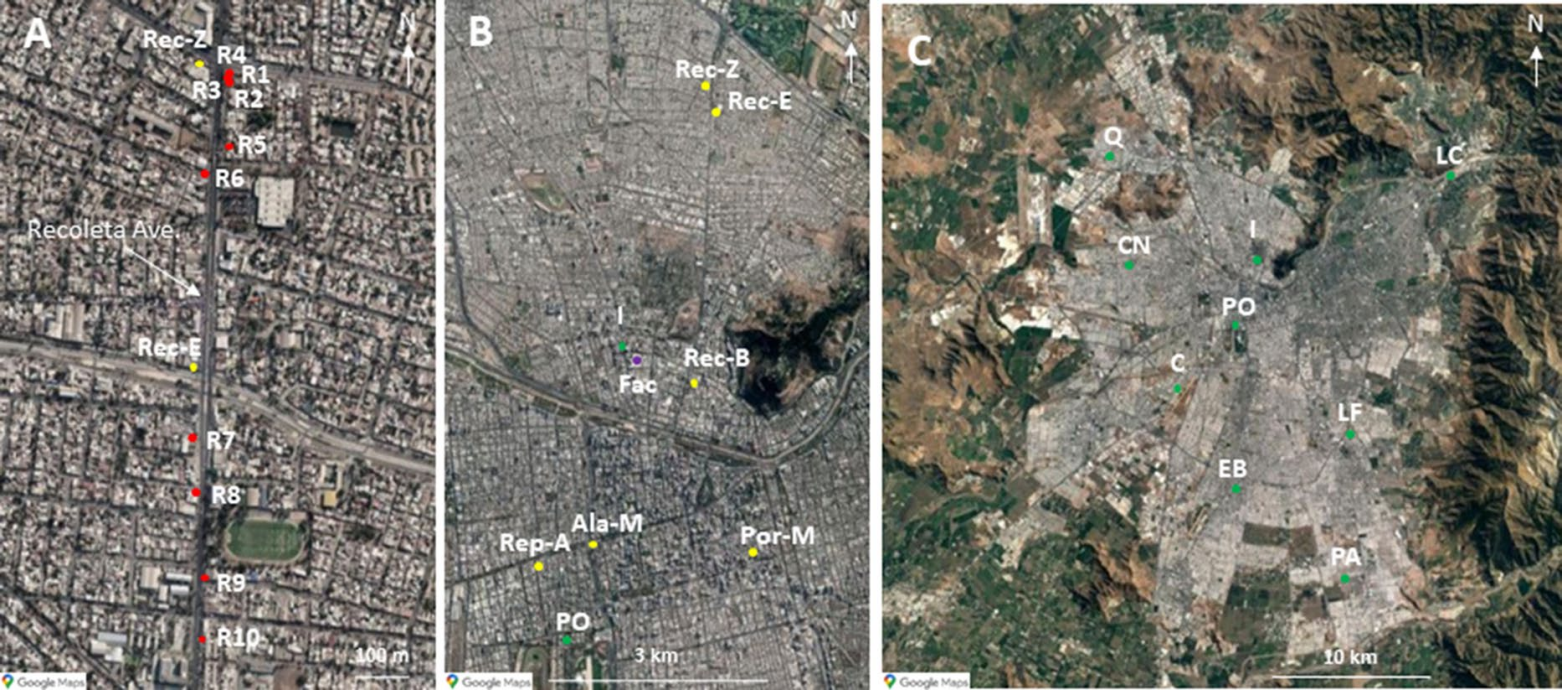
Samples group locations. **A** Recoleta sample group: location of ten specific *Acer negundo* individuals on Recoleta Avenue (red markers). **B** Mixed sample group: Location of eight urban tree species in Santiago. The locations of the intersections are: Rec-Z: Recoleta/Zapadores; Rec-E: Recoleta/Einstein; Rec-B: Recoleta/Buenos Aires; Ala-M: Alameda/Manuel Rodriguez; Rep-A: Republica/Alameda; Por-M: Portugal/Alameda. The Faculty site (Fac) is also indicated with a purple marker. **C** AOMS sample group: Location of nine *Quillaja saponaria* individuals installed in the Air Quality Monitoring Stations, Santiago. The locations of the sites are: PA: Puente Alto; EB: El Bosque; C: Cerrillos; PO: Parque O’Higgins; CN: Cerro Navia; LC: Las Condes, LF: La Florida; I: Independencia; Q: Quilicura

The 27 leaves samples and the 17 UD samples were organized into three groups (Fig. 1, Table SI-1): (A) Recoleta group corresponding to leaves of ten individuals of *Acer negundo* and UD along Recoleta Avenue (north of the capital), all sampled on the same day, at a distance between 0.6 and 1 m from the road. (B) Mixed tree species group corresponding to eight samples of *Olea europaea, Brachychiton populneus, Quillaja saponaria, Schinus molle, Cryptocarya alba, Acacia caven and Maytenus boaria* (one sample for each species), and the UD from streets and avenue in front of the trees, all sampled on the same day. (C). Air Official Monitoring Stations (AOMS) group corresponding to nine young individuals of *Quillaja saponaria* near nine AOMS (situated between 2 and 700 m from AOMS, but between 2 and 30 m from the street). They were sampled between November 30 and December 2, 2016, i.e., during the dry season.

*Acer negundo* is deciduous and has leaves only during austral spring–summer (October–March). *Olea europaea, Brachychiton populneus, Quillaja saponaria, Schinus molle, Cryptocarya alba, Acacia caven and Maytenus boaria* are permanent. Moreover, *Q. saponaria* renews its leaves every 2–3 years. Because all trees in the AOMS group were planted in November 2014, they all have undergone a similar exposure time of 2 years.

### Methods

#### Magnetic measurements

The magnetic measurements were carried out with two objectives: to quantify the amount of ferromagnetic minerals present in a sample and to obtain information about their nature and magnetic grain size. The first objective can be attained with two kinds of measurements. The bulk magnetic susceptibility (MS) is the magnetization acquired in a low field per unit field, normalized per weight. In first approximation, MS depends on the concentration of magnetic minerals and is widely used to quantify the amount of magnetic minerals. The Saturation Isothermal Remanent Magnetization (SIRM) is the remanent magnetization (i.e., measured in zero-field) that a sample acquires after being exposed to a magnetic field high enough to saturate its magnetization. Because the applied field is high, the SIRM values are able to quantify a magnetic contribution even when the magnetic minerals have a low concentration, as is the case in tree leaves.

The identification and magnetic grain size determination of magnetic minerals was addressed with the measurement of hysteresis loops, which is the variation of magnetization with an applied field (Fig. SI-1a). Three important parameters are extracted from the hysteresis loop and they can help identifying the bulk magnetic grain size: the saturation magnetization M_s_; the remanent magnetization M_r_ measured when the field is zero; the coercive field H_c_ which is the field necessary to bring the magnetization to a reversal. In addition, the measurement of the backfield curve gives access to the coercivity of remanence H_cr_, which is the field required to reach the point where the sample has zero remanence after the removing of the field (Fig. SI-1b). The combination of hysteresis parameters M_r_/M_s_ and H_cr_/H_c_ are used to construct a Day-plot (Day et al. 1977), which can then be used as a first approximation to determine an average magnetic grain size using experimentally and theoretically defined limits of the parameters (Fig. SI-1c). Another information is given by the acquisition of Isothermal Remanent Magnetization (IRM) as a function of applied field, since the field required to saturate a magnetic mineral can be different for different minerals (Fig. SI-1b).

MS measurements were carried out using AGICO KLY-3 Kappabridge at Institut de Physique du Globe, Paris. SIRM measurements were carried out using a Lakeshore Vibrating Sample Magnetometer (VSM) at the IMPMC-IPGP Magnetic Mineral Analysis Facility. A field of 1 T was applied, which is well-above the saturating field measured for all our samples. Hysteresis loops and IRM acquisition curves were also measured with the same instrument.

The values of MS and SIRM, normalized by mass, were then placed on a map with a color code according to their values, in order to obtain a cartography of the values.

#### PIXE measurements

Particle-induced X-ray emission (PIXE) is a technique for the elemental analysis of a sample, which is used as a target for the bombardment with a beam of accelerated particles; the interactions of the beam particles with the target atoms lead to the emission of X-rays of characteristic energies, through the detection of which the target composition can be deduced. In this technique, protons are almost universally chosen to induce X-ray emission (Lucarelli, 2020). *M. boaria, O. europaea* (in two sample sites), *Q. saponaria*, *S. molle, C. alba* and *B. populneus* leaves were analyzed for elemental composition. *M. boaria* and *O. europaea* samples were measured from two other sites: an urban street (Recoleta-Einstein) and a site within the Faculty. This latter site is on the Campus of the Faculty of Chemical and Pharmaceutical Sciences of University of Chile (33.3° lat. S and 70.4° long. W), located in Independencia commune in the city of Santiago; it has about 3 ha with small green areas and trees; it is surrounded by tall buildings reaching until 60 m (20 floors) and was considered as a site that should receive little exposure in PM. Leaves from *C. alba* were also sampled on this site. For each tree species, three leaves cleaned and three leaves exposed were analyzed by PIXE on both surfaces: adaxial (top) and abaxial (down). Leaves were washed following the method of Guerrero-Leiva et al. (2016). Elemental quantification was done using Micromatter standards (with a 5% uncertainty). Twenty-five elements were quantified on the samples: Na, Mg, Al, Si, P, S, Cl, K, Ca, Ti, V, Cr, Mn, Fe, Ni, Cu, Zn, As, Se, Br, Rb, Y, Zr, Mo, Pb.

The spectra acquired show that clean leaves are quite homogeneous, while the deposition of PM results in slight inhomogeneities that depend also on the leaves morphology such as veining and the side of the leaf analyzed (Fig. SI-2).

## Results

### Magnetic measurements on Recoleta sample group

**Fig. 2:**
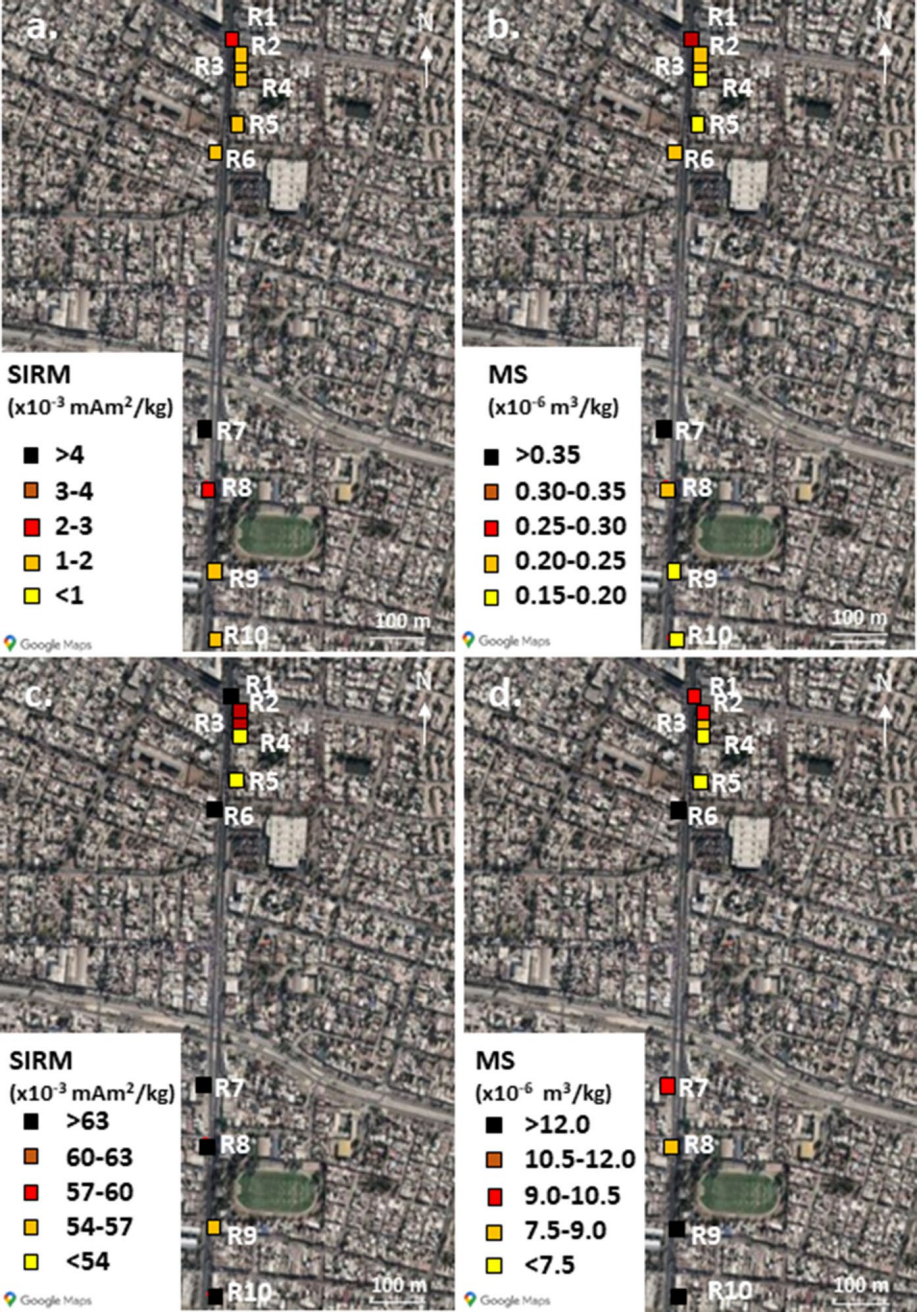
Cartography of magnetic parameters for leaves (**a**. SIRM; **b**. magnetic susceptibility) and urban dust (**c**. SIRM; d. magnetic susceptibility) for the samples taken along Recoleta Avenue (Recoleta sample group)

The magnetic properties of leaves of *A. negundo* and UD samples along Recoleta Avenue were measured. The SIRM and MS values for the leaves samples show a large variation, ranging from 1.7 to 4.3 × 10^–3^ mAm^2^/kg for the SIRM and from 0.17 to 0.44 × 10^–6^ m^3^/kg for the MS (Fig. 2a, b). For the UD samples, the SIRM values range from 45 to 85 × 10^–3^ mAm^2^/kg, and the MS values from 6.6 to 13.1 × 10^–6^ m^3^/kg (Fig. 2c, d).

**Fig. 3:**
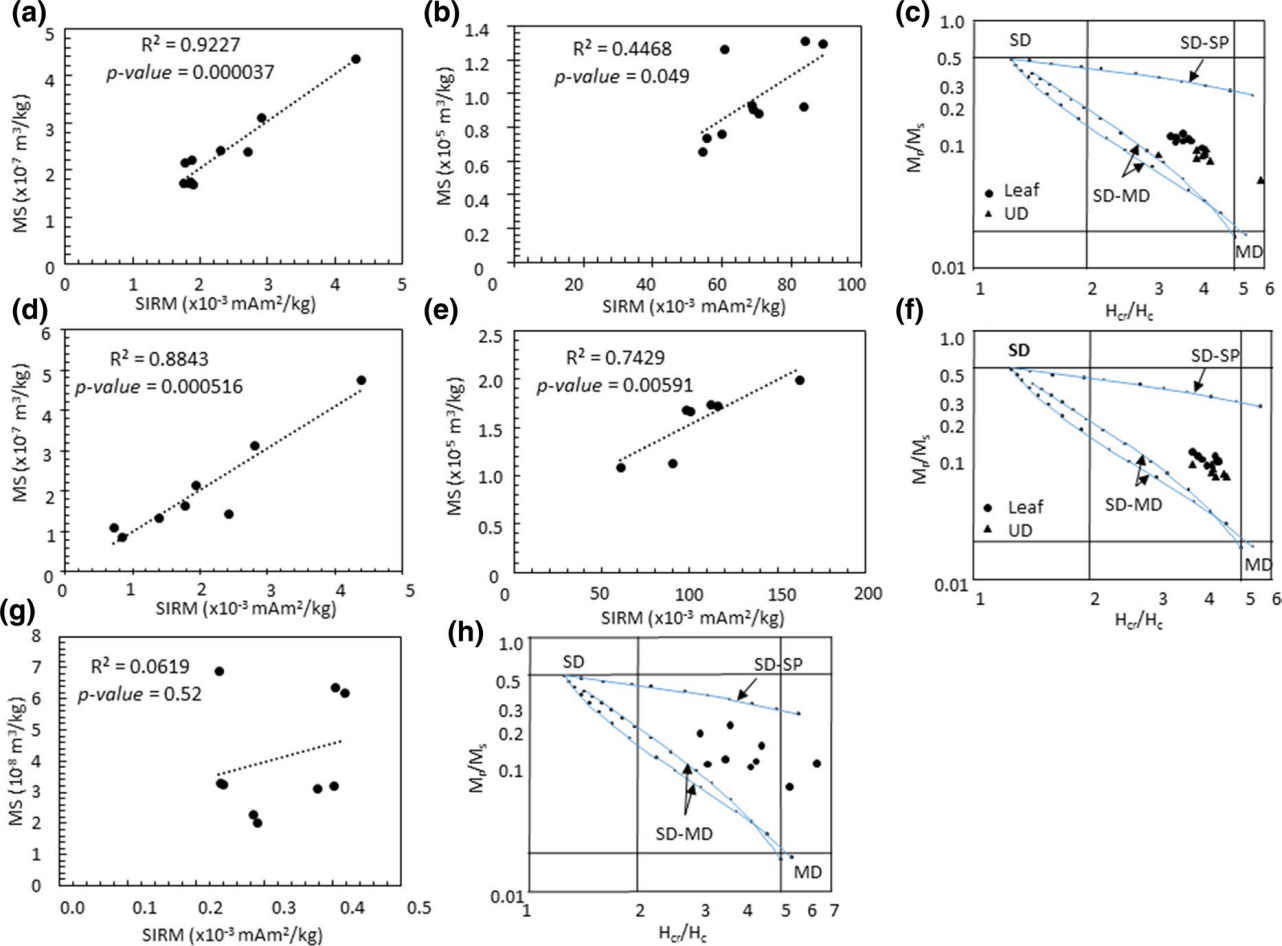
Magnetic properties of samples from Recoleta, Mixed and AOMS samples group. Correlation between SIRM and MS values for **a** leaf samples; **b** urban dust samples from Recoleta Avenue; **c** Day-plot for the Recoleta Avenue samples; Correlation between SIRM and MS values for **d** leaf samples; **e** urban dust samples from the mixed species samples; **f** Day-plot with the mixing lines from Dunlop (2002) for the Mixed Species samples; **g** correlation between SIRM and MS values for leaf samples from AOMS sample group; **h** DDay-plot with the mixing lines from Dunlop (2002) for the AOMS sample group

There is a good correlation between SIRM and MS values for the leaf samples (*p-value* = 0.000037), indicating that the magnetic mineralogy is similar within the sample group (Fig. 3a). The correlation is less good for the UD samples (*p-value* = 0.049) (Fig. 3b). The similar magnetic mineralogy is further confirmed by Day-plots: the hysteresis parameters are clustered in the pseudo-single-domain (PSD, see Fig. SI-1)) region, with the UD samples slightly closer to the multidomain (MD) region than the leaf samples, and slightly more dispersed (Fig. 3c). This indicates that the magnetic grain size is the same for all the leaf samples and the UD samples to a lesser extent, respectively. It also shows that this magnetic grain size is slightly larger for UD samples than for leaf samples.

The SIRM acquisition curves (Fig. SI-3) show that the SIRM is reached for all samples when the applied field is around 0.3 T or lower. These values < 0.3 T are characteristic for magnetite-like minerals, which are the expected Fe-rich particles from a vehicular source (e.g., Gonet & Maher, 2019; Mitchell & Maher, 2009), and indicate that high-coercivity minerals such as hematite are absent.

### Magnetic particles and elemental composition on Mixed sample group

The magnetic properties of leaves of *O. europaea, S. molle, A. caven, M. boaria, C. alba, B. populneus and Q. saponaria* leaves and UD samples were measured. Because the leaves come from trees of different species, the spatial cartography for this group of samples cannot be interpreted as a spatial pollution monitoring tool, but it could be a useful screening tool. We measure SIRM values between 0.7 and 4.3 × 10^–3^ mAm^2^/kg and MS values between 0.08 and 0.48 × 10^–6^ m^3^/kg for the leave samples (Fig. SI-4a and b). For the corresponding UD samples, the SIRM values range from 60 to 163 × 10^–3^ mAm^2^/kg and the MS values from 10.94 to 20.02 × 10^–6^ m^3^/kg (Fig. SI-4c and d). These values are in the same order of magnitude as for the Recoleta Avenue samples group. The highest SIRM and MS values are obtained for *O. europaea*, *S. molle* and *A. caven* leaves, while those values are much lower for *M. boaria, C. alba, B. populneus* and *Q. saponaria* leaves.

The good correlation between SIRM and MS for all tree or UD samples (Fig. 3d, e), as well as the similar hysteresis parameters which plot in the same area on the Day-plot (Fig. 3f) suggest that the magnetic mineralogy is consistent within the sample set (*p-value* = 0.000516 for the leaf samples and 0.00591 for the UD). The leaves hysteresis parameters are slightly closer to the SD region than the UD parameters.

The SIRM acquisition curves show the same saturation field for all leaves and UD samples around 0.3 T or lower, which is consistent with a magnetite-like value (Fig. SI-5). When comparing the SIRM or MS of leaves with those of UD, we find that there is no correlation between both, indicating again that the magnetic signal in UD and leaves do not have the same contributions, even though the magnetic minerals have almost the same magnetic signature (Fig. SI-6).

**Fig. 4:**
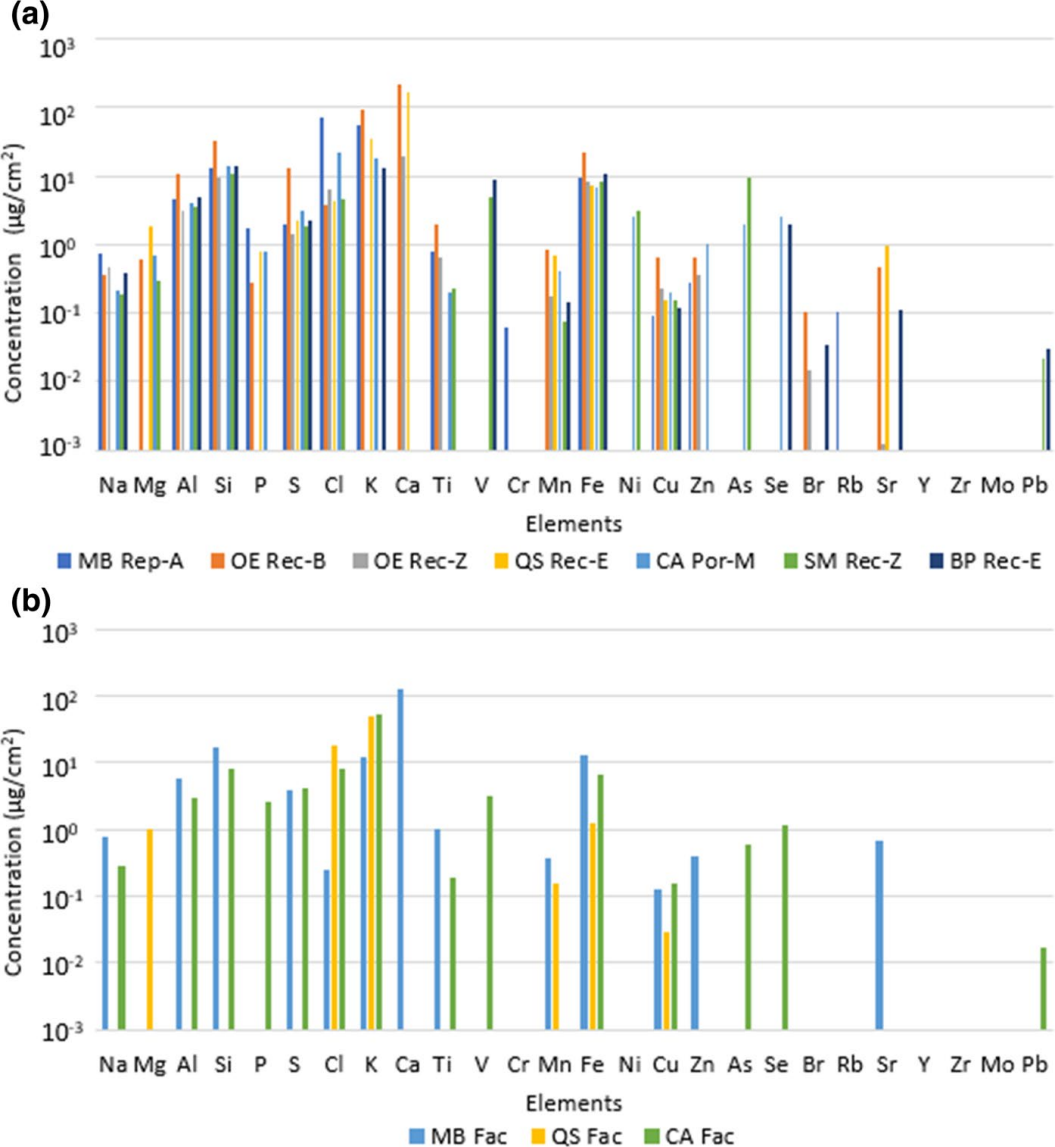
Elemental concentration on: **a** leaves of *Maytenus boaria* at Republica/Alameda (MB Rep-A), *Olea europaea* at Recoleta/Buenos Aires (OE Rec-B) and at Recoleta/Zapadores (OE Rec-Z), *Quillaja saponaria* at Recoleta/Einstein (QS rec-E), *Cryptocarya alba* at Portugal/Marin (CA Por-M), *Schinus molle* at Recoleta/Zapadores (SM Rec-Z), *and Brachychiton populneus* at Recoleta-Einstein (BP Rec-E); **b** *Maytenus boaria* (MB Fac)*, Quillaja saponaria* (QS Fac) and *Cryptocarya alba* (CA Fac) leaves on Faculty site

In general, *O. europaea* (Rec-B), *Q. saponaria* (Rec-E) and *M. boaria* (Rep-A) leaves present the highest elemental concentration (Fig. 4a). The most predominant elements for most of the species are Na, Al, Si, S, Cl, K, Mn, Fe, Cu and Zn on urban street. Potassium, Cl, Fe and Cu are observed in the three species on Faculty (Fig. 4b). The total elemental concentration for *O. europaea* leaves at the Rec-B site is 385 μg/cm^2^, while that of leaves of the same species at the Rec-Z site is only 30 μg/cm^2^, i.e. the Rec-B site collected ten times more PM (mainly Ca, K and Si) than the Rec-Z site (Si, Fe and Cl). Magnesium, Al, Si, P, S, Cl, K, Ca, Ti, Mn, Fe, Cu, Br and Zn predominate in the abaxial side of *O. europaea* at Rec-B site, while at Rec-Z site, S and Ca predominate in the adaxial side and Ti, Mn, Fe and Br predominate in the abaxial side of the leaves.

**Fig. 5:**
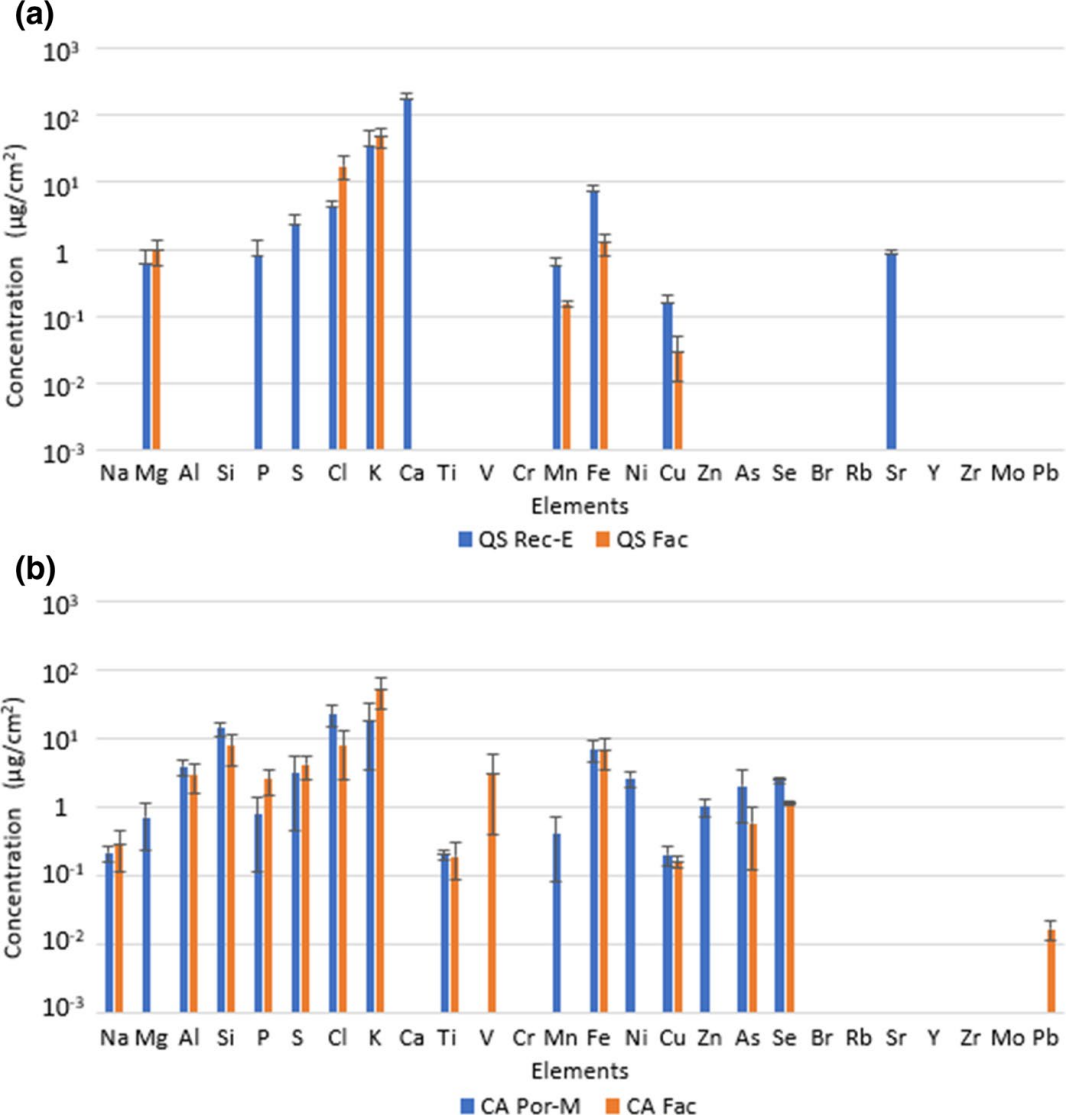
Elemental concentration and standard deviation for: **a** *Quillaja saponaria* (QS) leaves at Recoleta-Einstein (Rec-E) and Faculty (Fac) sites; **b** *Cryptocarya alba* (CA) leaves at Portugal-Marin (Por-M) and Faculty (Fac) sites

*M. boaria* leaves present a total elemental concentration of 159 μg/cm^2^ at Rep-A site and 177 μg/cm^2^ on the Faculty sites. Chlorine, K and Si have the highest concentrations on Rep-A site leaves, while Ca, Si and Fe are predominant on leaves from Faculty site. Aluminum, Si, Ti, Fe, Cu and Zn are the elements that are the most concentrated on the adaxial side of *M. boaria* leaves from Rep-A site, while Al, Si, K, Ti, Fe and Cu are the most concentrated on the abaxial side of *M. boaria* leaves from Faculty. *Q. saponaria* leaves have a concentration of 225 μg/cm^2^ and 69 μg/cm^2^ at Rec-E site and Faculty site, respectively. The elements Ca, K and Fe are the most concentrated in Rec-E and K, Cl and Fe in Faculty (Fig. 5a). Manganese and Fe are much more concentrated on the abaxial side of leaves from Rec-E, while Mg and K are in higher concentration on the adaxial side. Manganese and Fe are higher on the abaxial side, and Cl and Cu are present on both sides of leaves from Faculty. *C. alba* leaves presented the same deposit at Por-M site as in Faculty 79 μg/cm^2^ and 89 μg/cm^2^, respectively. Potassium, Cl and Si are the most concentrated at Por-M site and Faculty (Fig. 5b). Magnesium, Al, Si, P, S, Cl, K, Mn, Fe, Ni, Cu, Zn and Se are on both sides of leaves in Rec-E, while, Al, Si, P, Cl, K, Fe and Cu on Faculty.

*S. molle* (Rec-Z site) and *B. populneus* (Rec-E site) leaves had a total elemental concentration of 48 μg/cm^2^ and 58 μg/cm^2^, respectively. In *S. molle,* Si, As and Fe were the most predominant elements (Fig. 4a), Sodium, Si, Cu, As and Pb are in higher concentration on the abaxial side and Mg, S, Cl, Ti, V, Mn and Ni are higher on the adaxial side. Aluminum is similar on both sides. In *B. populneus* Si, K and Fe were the most predominant elements, Na, Al, Si, Fe, Cu, Br and Pb are in higher concentration on the abaxial side of leaves, K and Se are predominant on the adaxial side, and S, V, Mn and Sr are similar on both sides.

### Air official monitoring stations (AOMS) group

**Fig. 6:**
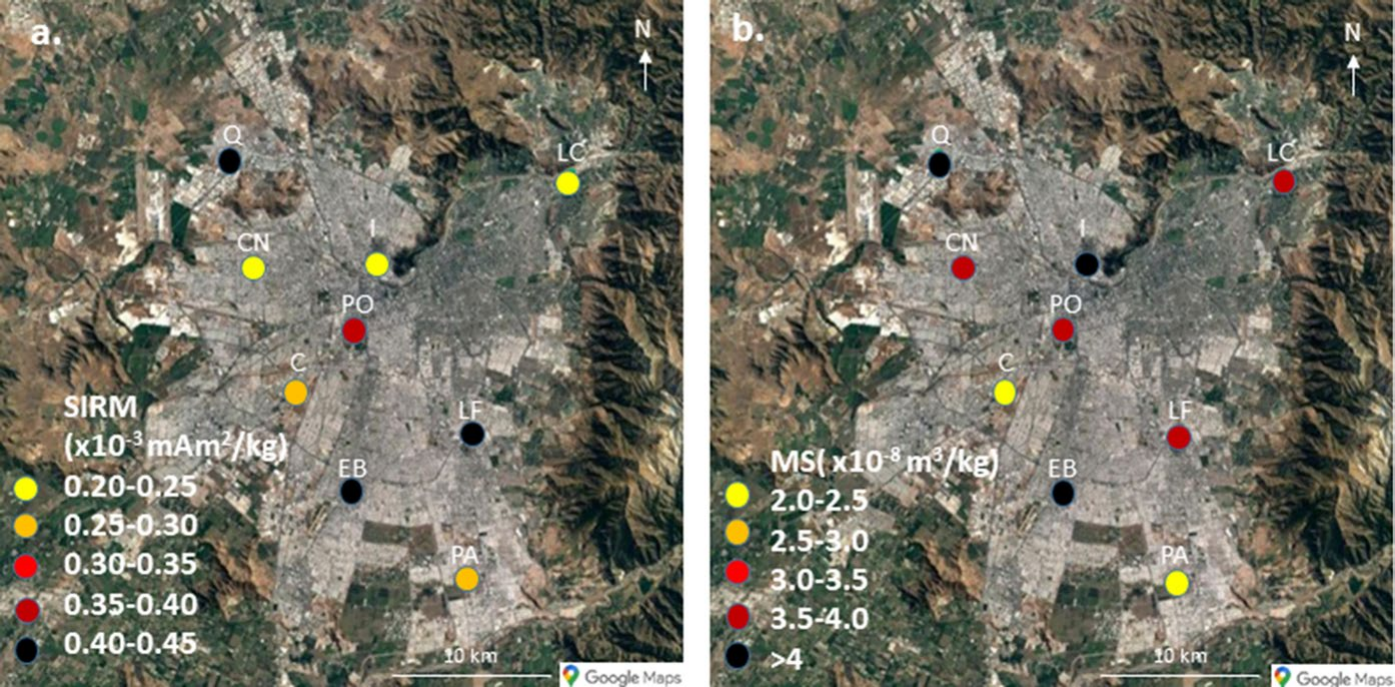
Cartography of magnetic parameters for leaves from AOMS sample group (**a**. SIRM; **b**. magnetic susceptibility)

Only *Q. saponaria* leaves were sampled for this sample set, close to AOMS distributed throughout Santiago. SIRM and MS values are very weak compared with values from the other sample sets and other similar studies. The SIRM values range between 0.20 and 0.42 × 10^–3^ mAm^2^/kg, while the MS values range between 0.02 and 0.07 × 10^–6^ m^3^/kg (Fig. 6).

Unlike the previous sample sets, there is no correlation between MS and SIRM values (*p-value* = 0.52), showing that the magnetic mineralogy is different within the sample set (Fig. 3g). This is confirmed by the hysteresis parameters that are much more dispersed than in the previous data sets, though mostly in the PSD region (Fig. 3h). SIRM acquisition curves are fairly noisy, since the magnetic signal is very weak, nevertheless the saturation still happens around 0.3 T and shows a predominance of a magnetite-like magnetization carrier (Fig. SI-7).

## Discussion

### General remarks about magnetic properties

All samples (leaves and UD) have a magnetic mineralogy consistent with magnetite-like minerals in the PSD size range. This agrees with most other studies that used passive captors as urban pollution proxy (e.g., Maher et al., 2008; Mitchell & Maher, 2009). The values recorded in leaves through MS and SIRM are several times higher than the values measured by Muñoz et al. (2017) in Santiago in *Platanus x acerifolia*, but the area covered in that study was probably traveled by newer private vehicles that emit less PM than the busier areas sampled in this study. For UD samples, MS and SIRM values are higher than the values of Aguilar et al. (2012) measured in Bogota, but the same order of magnitude as the values of Muñoz et al. (2017), this time in the same area of Santiago than the one studied here. Indeed, in Muñoz et al. (2017), two different type of communes, corresponding to two different environments, were studied: one with newer private vehicles and less public vehicles and the other one similar to group A of this study.

The Recoleta and the mixed tree species sample sets also show that the magnetic particles deposited on tree leaves have the same origin, unlike those found in soil. This could be caused by the fact that the magnetic signal in UD also has a natural origin that is not so important on tree leaves, and reinforces the conclusion that the leaves are better indicators of anthropogenic magnetic PM than UD. The particles from the exhaust pipe of motor vehicles are mainly found in the fine fraction of the PM, and therefore, they are more easily deposited at a higher altitude than the larger particles that are more abundant in the soil.

### Recoleta group

The values measured in UD samples are the same order of magnitude as those measured in Recoleta sector by Muñoz et al. (2017). The profile along Recoleta Avenue in Las Condes commune (*A. negundo*) is useful to identify PM hotspots. For instance, the high values measured at the point R1, close to the subway station, can be explained by the presence of a major intersection coupled with a bus hub. At point R7, the presence of a car repair shop could cause the high values observed in this area. The results for the UD samples are quite consistent with the results for the leaves. Urban dust collected at sites R1 and R7 also show high values of SIRM. This sample set shows that important information could be gained from this measurement type and could be used to issue recommendations for pedestrians. For instance, if such measurements are carried out on sidewalk both sides of a street, it could lead to information as simple as which side of the road is the best to walk on to minimize PM the exposition.

### Mixed species group

The leaf samples show that in decreasing order, *O. europaea* presents a high magnetic signal located at the Rec-B site, followed by *S. molle* and *A. negundo* that are at the Rec-Z site, and followed by *A. caven* located at the Ala-M site. All those streets have high vehicular traffic (highest in Alameda). The values obtained with the *O. europaea* sampled at the Rec-Z site are weak, compared to those obtained from *S. molle* at the same location and *O. europaea* at Rec-B site; however, the signal from *A. negundo* coincides with that recorded by *S. molle*.

In addition, samples of this group were analyzed by PIXE, providing the first background of elemental composition leaves by this technique. Various elements were identified on both sides of the sample leaves: Na, Al, Si, S, P, Cl, K, Ca, Ti, Cr, Mn, Fe, Cu, Zn, Rb, Mo and Pb. The identification of Fe is consistent with the magnetic particle signals indicated. In the leaves samples from the Faculty site (closed space), a lower proportion of some elements was observed. In general, the leaves of *O. europaea* from the Rec-B site, *Q. saponaria* (Rec-E) and *M. boaria* (Rep-A) presented a higher proportion of elements per surface area. This can be related to a greater proximity to the vehicular traffic, which is the main source of emission, and to the properties of the species that contribute to a greater capture of particles.

The high elemental concentration in *M. boaria* in the Faculty site could be explained by its proximity to a construction site of a mall and a building, which could also explain the high Ca concentration. *C. alba* leaves from the Faculty site shows similar elemental concentration compared to the leaves from Por-M site.

There are two sites in this group of samples where two different trees are co-located: 1) *S. molle* and *O. europaea* (Rec-Z) collecting a different concentration of PM with a different elemental composition and distribution of the element on both sides of the leaves; however, both collect heavy metal as As, Mn, Fe or Pb concentrated on the abaxial side. Magnetic parameters have higher values for *S. molle* leaves than for *O. European* leaves, which seems consistent with the PM concentration data. 2) *Q. saponaria* and *B. populneus* also collect very different concentration of PM and both collect the heavy metals as Mn, Fe, Cu or Pb concentrated on the abaxial side of leaves. The consistency of the elemental results with magnetic parameters is not as good, since they are very similar for the leaves from both trees. These results show that the ability to retain PM is species-specific.

The comparison between concentrations on the abaxial and adaxial sides shows that in general, elemental concentrations are higher in the adaxial side. In particular, concentrations of elements which are mainly in the coarse fraction (like crustal elements Al and Si, and abrasion element, such as Fe) are higher in the adaxial side, which is compatible with a major contribution of settling as removal process, while elements which are mainly in the fine fraction, such as S (secondary particulate produced from SO_2_), are found on both sides, which is compatible with a major contribution of diffusion.

The elements detected have been previously identified in PM_10_ and PM_2.5_ in cities. Some of them are mainly linked to windblown soil resuspension or dust transport (e.g., Al, Si, Ti…), others to anthropogenic emissions (e.g., Cu and Zn from vehicular brake and tire wear, dust from traffic resuspension). Pattanaik et al. (2020) indicate that Zn as its speciation, coming from residual oil combustion, varies through the different size fractions of PM and has implications on the bioavailability and toxicity of Zn. Although some elements are specific markers for some sources, most elements may have contributions from different sources and the quantification of such contributions may be carried out only by using source apportionment studies (Amato et al., 2016; Lucarelli et al., 2015). The natural fraction always corresponds to the principal one, so the natural elements present greater concentrations in soils, urban dust or atmospheric aerosols. The anthropogenic elements are trace elements and their concentrations are smaller. This trend was observed in the particulate matter deposited on leaves (Fig. 5). For instance, Na (marine aerosols), Al, Si, Ca and Ti have a terrestrial crust origin, while S, P, Cl, K, Cr, Mn, Fe, Cu, Zn, Rb, Mo and Pb are anthropogenic; additionally, Mg, V, Cr, Mn, Fe, Ni, Cu, Zn, As and Pb have been identified as coming from different industrial processes (Préndez et al., 2007). Lead is still present in the atmosphere coming from the combustion of lead gasoline, as well as Br.

We looked for possible correlations between the magnetic parameters (magnetic susceptibility and SIRM) and the elemental concentrations. The correlation between total elemental concentrations and magnetic parameters is weak (*R*^*2*^ = 0.36 for SIRM and 0.42 for MS) and not significant at *p* < 0.05 (*p-value* = 0.15 for SIRM and 0.12 for MS). Individual elements that are associated with magnetic PM are Zn, Cd, Pb, Ni, Cu and Cr (Cao et al., 2015). Here, only Cu shows a weak correlation with magnetic parameters (*R*^*2*^ = 0.49 for SIRM and 0.61 for MS; *p-value* = 0.08 for SIRM and 0.04 for MS); the other elements cited by Cao et al. (2015) either show no correlation with magnetic parameters or were not measured in enough leaves samples to enable a meaningful correlation analysis. The other elements that are correlated with magnetic parameters are S (*R*^*2*^ = 0.64 for SIRM and 0.70 for MS; *p-value* = 0.02 both for SIRM and MS) and Br (*R*^*2*^ = 0.83 for SIRM and 0.51 for MS, but *p-value* = 0.09 for SIRM and 0.29 for MS).

### AOMS group

The magnetic values obtained for all *Q. saponaria* leaves at AOMS have much lower values (almost an order of magnitude) than those of all other samples measured. We only have one value other than from this sample set for a *Q. saponaria* sample, and it is much higher than the AOMS values. There might be an effect of the age of the individual trees that can affect the capture of PM (Préndez et al., 2013): the *Q. saponaria* in the AOMS group are young compared to the other *Q. saponaria*. But it could also suggest that the station locations might not be representative of the actual PM pollution that most of the town of Santiago experiences, at least from traffic pollution. The magnetic particles present on the leaves do not seem to have all the same origin as in the two other sample sets; since the sampling sites are slightly further away from the roads, this may be an effect of a larger diversity of sources of the anthropogenic component that would not be limited to vehicular emissions but also industrial emissions or burning of biomass, or an effect of the smaller anthropogenic component, which makes the natural component more visible; this component has a magnetic signature more diverse than the anthropogenic component.

Because the PM that are responsible for the magnetic signal are deposited as well as integrated inside the leaves, rainfall can affect the magnetic response, removing deposited PM on their surface by washing (Castaneda-Miranda et al., 2020; Matzka & Maher, 1999; Mitchell & Maher, 2009). The rainy season in Santiago takes place from April until September, so outside of the sampling period. Moreover, we did not try to compare the different values. Rather, we obtained information about the order of magnitude in the magnetic signal and information about the diversity of provenance of magnetic PM; these two pieces of information are less affected by rainfalls than a true quantitative comparison between sites.

In order to extend these observations to the level of individual trees, the foliar surface of each tree must be considered. Calculations of the Leaf Area Index (LAI) of individual trees cover are in progress, in order to estimate the effective removal of PM from vehicle emissions by different tree species in Santiago.

Similar studies about the mitigating function of trees could be replicated in other cities of Chile, keeping in mind other criteria such as restriction/abundance of soil, or water and space conditions. Such studies will help in the elaboration of strategies for future urban climate scenarios.

## Conclusions

We have carried out a magnetic and elemental study on tree leaves as well as a magnetic study on UD in Santiago, Chile. The main results are the following:

- Magnetic signal measured on urban tree leaves is a good proxy for tracing anthropogenic metallic particles, while similar measurements on urban soil may be more influenced by particles of detritic (natural) origin.
- Magnetic measurements on tree leaves can be used in spatially localized studies in cities to help identify PM hotspots, thanks to the excellent spatial resolution of this technique.
- Elemental PIXE analysis of tree leaves allows to quantify many elements associated with vehicular emissions. Copper, Zn, Fe, K and S are present on every site, while As, Se, V, Ni, Sr, Zr, Mo and Pb are identified on some sites. This technique can also distinguish the elemental composition on both sides of the leaves.
- The results obtained reinforce the role of trees as specie-specific biomonitors in the capture of PM in terms of quantity and quality of the material removed from the atmosphere, depending on the environment surrounding the plant.

## Supplementary Information

Below is the link to the electronic supplementary material.

Supplementary file1 (DOCX 3878 KB)

## Data Availability

The datasets generated during and/or analyzed during the current study are available from the corresponding author on reasonable request.
