# Supplementary material for: Magnetic and elemental characterization of the particulate matter deposited on leaves of urban trees in Santiago, Chile
Source: Environ Geochem Health. 2022 Sep 6;45(5):2629–43. doi: 10.1007/s10653-022-01367-w (PMC10140104; doi:10.1007/s10653-022-01367-w)
Supplement: Supplementary file 1 — Supplementary file1 (DOCX 3878 KB) [file 10653_2022_1367_MOESM1_ESM.docx]

**MAGNETIC AND ELEMENTAL CHARACTERIZATION OF THE PARTICULATE MATTER DEPOSITED ON LEAVES OF URBAN TREES IN SANTIAGO, CHILE.**

M. Préndez^1^, C. Carvallo* ^2^, N. Godoy^1^, C. Egas^3^, B.O. Aguilar Reyes^4^, G. Calzolai ^5^, R. Fuentealba^1^, F. Lucarelli^5^, S. Nava^5^

**Detail on sampling and methods:**

Sampling: Urban dust (UD) was swept from the asphalt with a bristle brush from a delimited area of 1 m^2^ near the leaf-sampling site, and subsequently stored in sterile bags until analysis. In the laboratory, the leaves were dried at 40 °C for 12 h and then grinded; the UD samples were sieved through 600 μm sieve. Both types of samples were weighed and packaged into acrylic boxes (8 cm^3^ of internal volume) to carry out the magnetic and the elemental composition analysis.

PIXE measurements: To clean leaves, they were carefully brushed and rinsed with ultra-pure water to assess the “blank leaf” composition on which PM is deposited. Five points were measured on every surface leaves to average possible sample not-homogeneity. Every point was measured during 60s, using an extracted in air 3 MeV proton beam (area: of 1 mm x 2 mm), the same fully dedicated to aerosol measurements at the INFN-LABEC laboratory in Florence. The set-up counts on three Silicon Drift Detectors (SDD) as comprehensively described in (Calzolai et al. 2015; Lucarelli et al. 2018; Lucarelli 2020). PIXE spectra were fitted using the GUPIXW in software (Campbell et al. 2010).

References:

Calzolai, G., Lucarelli, F., Chiari, M., Nava, S., Giannoni, M., Carraresi, L., Prati, P., Vecchi, R. : Improvements in PIXE analysis of hourly particulate matter samples. Nuclear Instruments and Methods in Physics Research Section B: Beam Interactions with Materials and Atoms 363, 99-104 (2015). <https://doi.org/10.1016/j.nimb.2015.08.022>

Campbell, J.L., Boyd, N.I., Grassi, N., Bonnick, P. & Maxwell, J.A.: The Guelph PIXE Software Package IV. Nuclear Instruments and Methods in Physics Research Section B: Beam Interactions with Materials and Atoms 268, 20, 3356–3363 (2010). <https://doi.org/10.1016/j.nimb.2010.07.012>

Lucarelli, F., Calzolai, G., Chiari, M., Nava, S., Carraresi, S.: Study of atmospheric aerosols by IBA techniques: The LABEC experience. Nuclear Instruments and Methods in Physics Research Section B: Beam Interactions with Materials and Atoms 417,121-127 (2018). <https://doi.org/10.1016/j.nimb.2017.07.034>

Lucarelli, F.: [How a small accelerator can be useful for interdisciplinary applications: the study of air pollution](https://www.scopus.com/record/display.uri?eid=2-s2.0-85087357454&origin=resultslist). The [European Physical Journal Plus](https://www.scopus.com/sourceid/21100201754?origin=resultslist), 135, 7, 538 (2020). <https://doi.org/10.1140/epjp/s13360-020-00516-3>


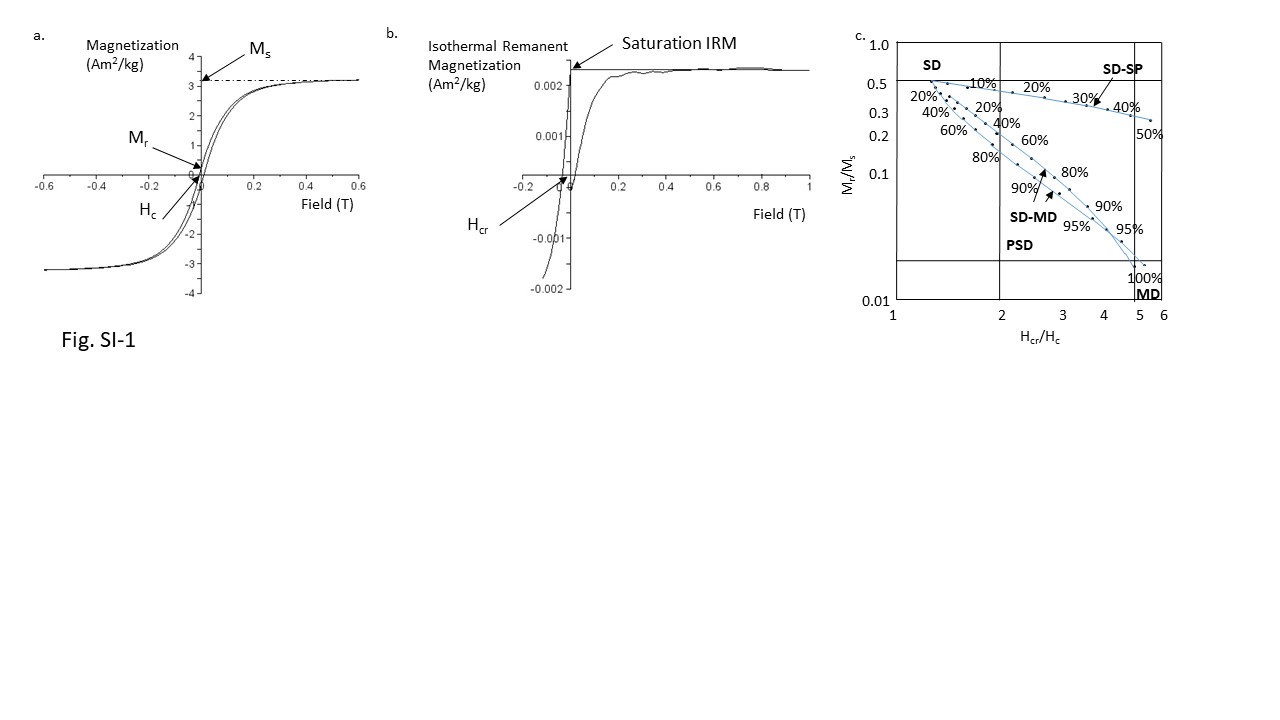


**Fig. SI-1** Definition of hysteresis parameters. a. Hysteresis loop; b. Backfield and IRM acquisition curve; c: Magnetic grain size identification using a Day-plot with the mixing lines from Dunlop (2002)

**
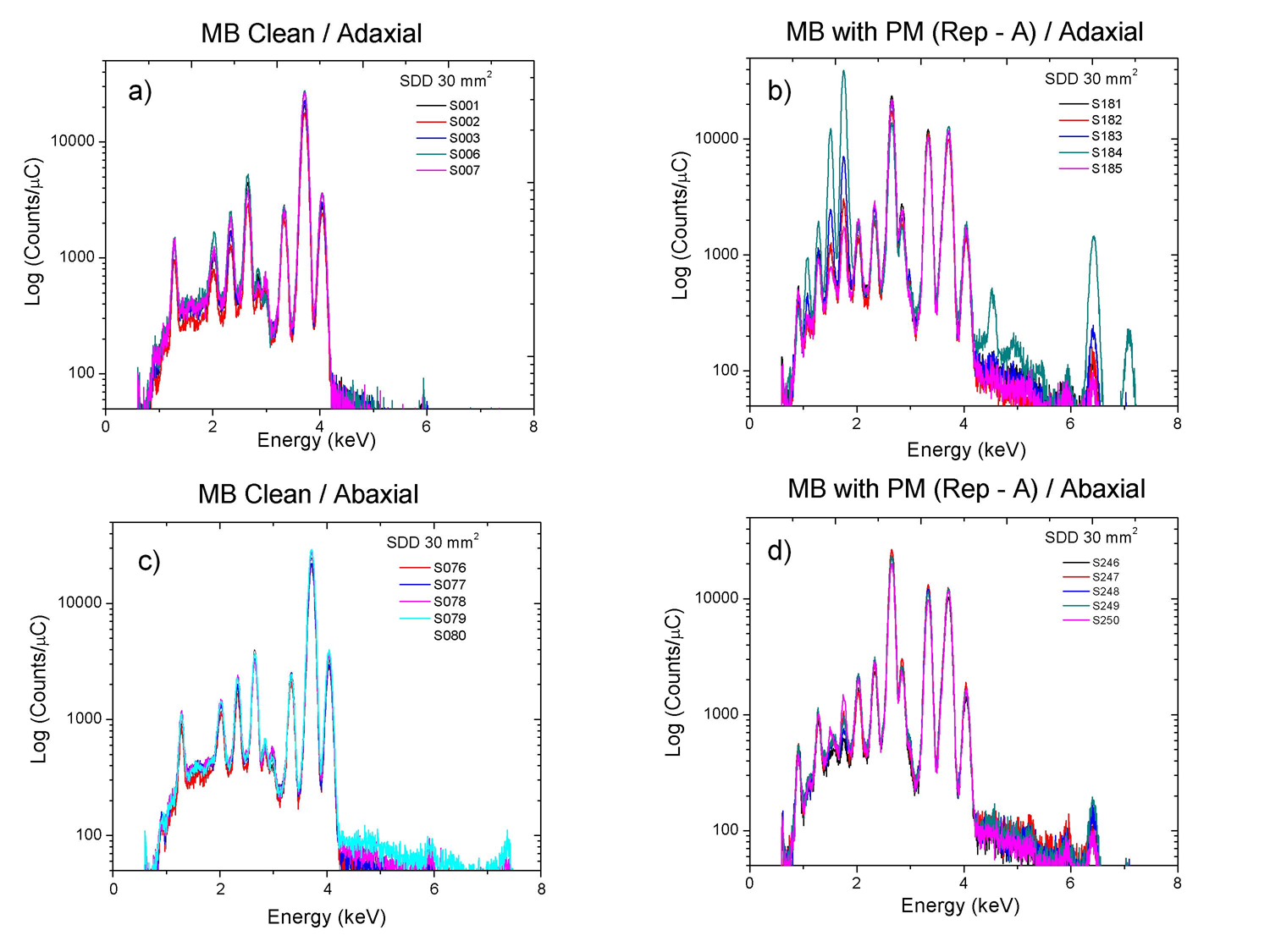
**

**Fig. SI-2** PIXE spectra of a *Maytenus boaria* (MB) leaf sample, obtained with SDD 30 mm^2^ for low-Z element detection: a) clean adaxial side, b) adaxial side with particulate matter, c) clean abaxial side, d) abaxial side with particulate matter.


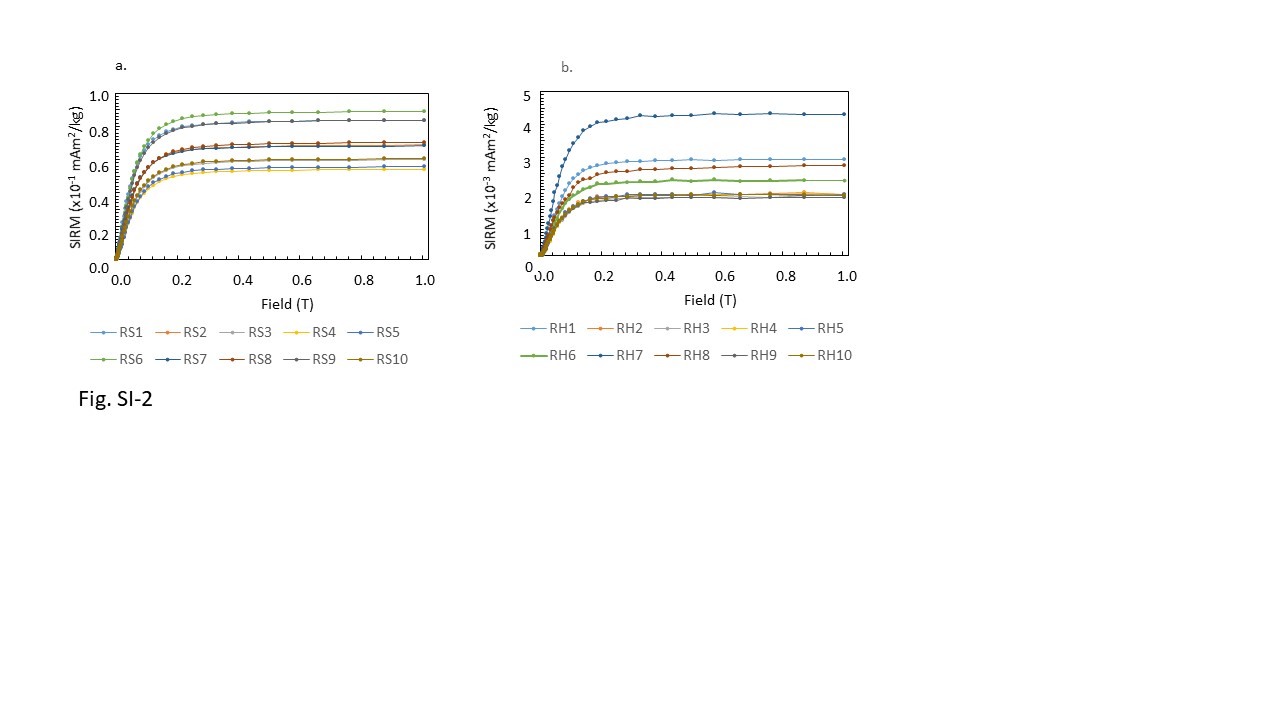


**Fig. SI-3** SIRM acquisition curves for samples from Recoleta avenue. a: Leaves; b: Urban dust

**
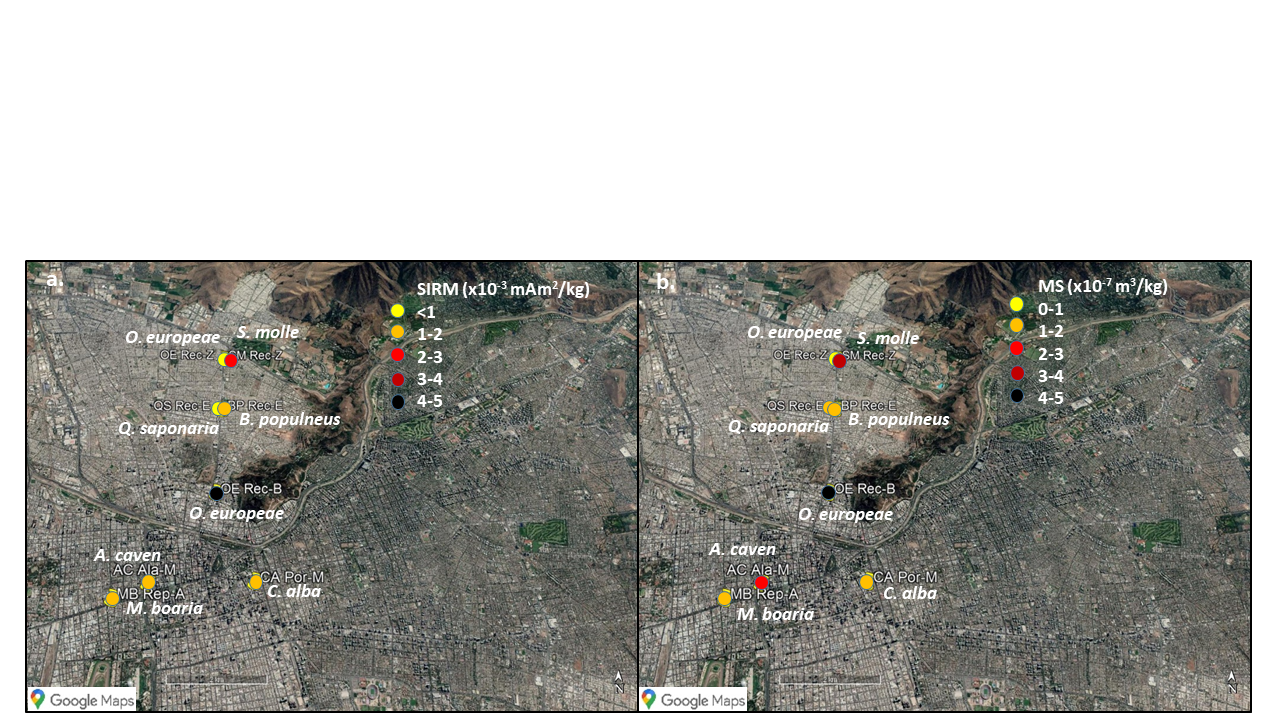
**

**
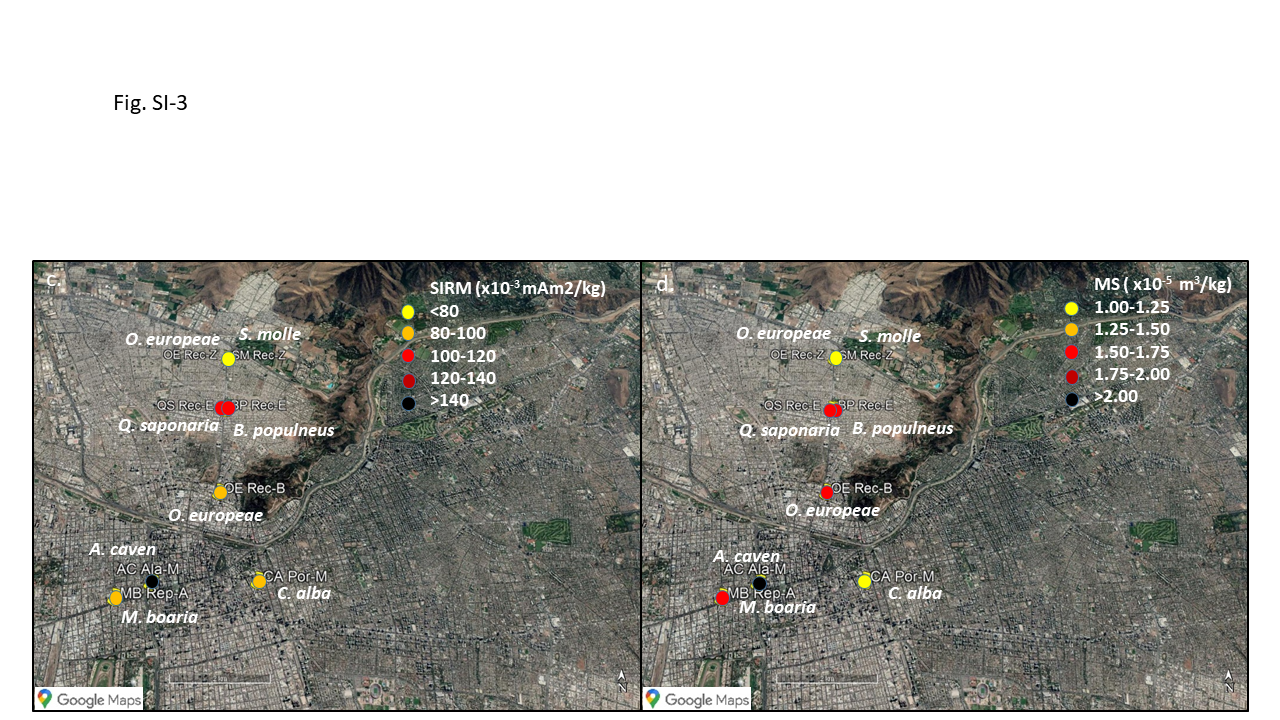
**

**Fig. SI-4** Cartography of magnetic parameters for leaves (a. SIRM; b. magnetic susceptibility) and urban dust (c. SIRM; d. magnetic susceptibility) for the samples from various tree species.


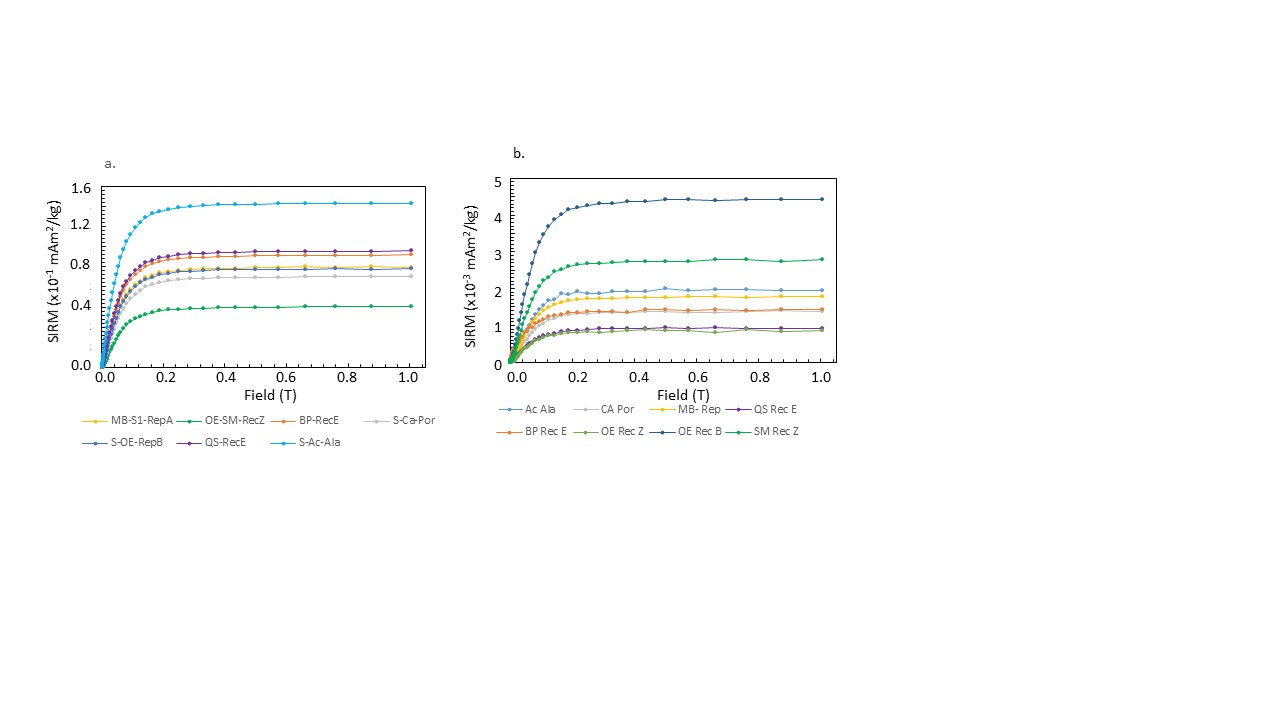


**Fig. SI-5** SIRM acquisition curves for samples from the various tree species. a: Leaves; b: Urban dust


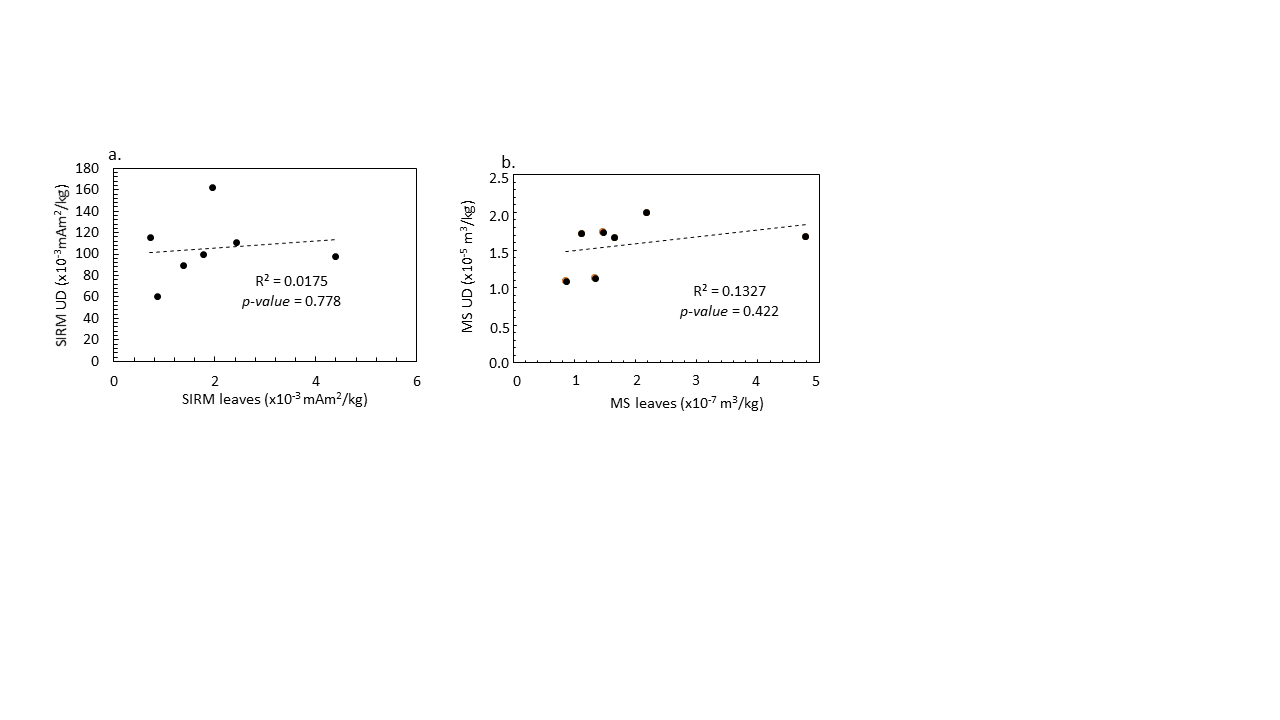


**Fig. SI-6** Correlation between a: SIRM of soils and leaves, and b: MS of urban dust and leaves for the Mixed Species sample set


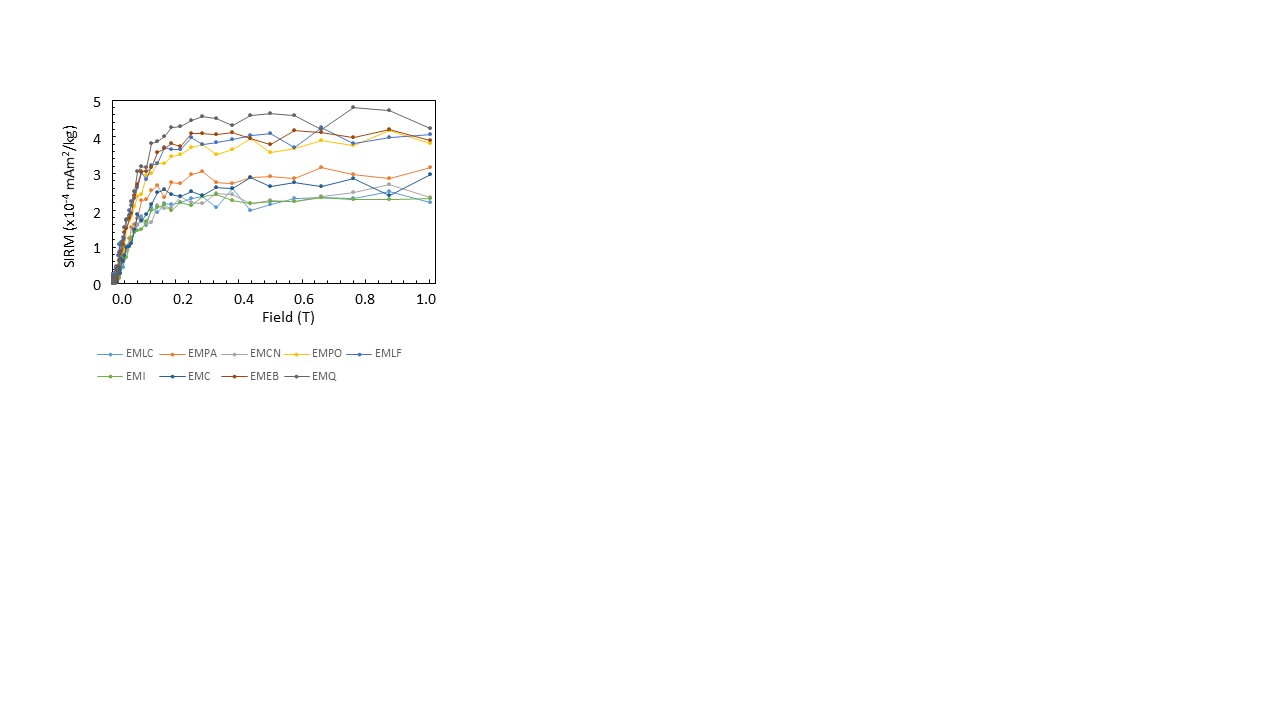


**Fig. SI-7** SIRM acquisition curves for samples from leaves from air quality monitoring stations

Table SI-1: Sampling sites, species and sample names of the leaves and urban dust sampled and used for magnetic measurements.

| **Urban tree species** | **Sampling Sites** | **Month-year** | **Leaves Label** | **Weight (g)** | **Soils Labels** | **Weight (g)** |
| --- | --- | --- | --- | --- | --- | --- |
| 1. **Recoleta sample Group** | | | | | | |
| *Acer negundo* | Av. Recoleta | Nov-15 | RH1 | 0.138 | RS1 | 0.404 |
| *Acer negundo* | Av. Recoleta | Nov-15 | RH2 | 0.130 | RS2 | 0.376 |
| *Acer negundo* | Av. Recoleta | Nov-15 | RH3 | 0.124 | RS3 | 0.453 |
| *Acer negundo* | Av. Recoleta | Nov-15 | RH4 | 0.120 | RS4 | 0.406 |
| *Acer negundo* | Av. Recoleta | Nov-15 | RH5 | 0.110 | RS5 | 0.339 |
| *Acer negundo* | Av. Recoleta | Nov-15 | RH6 | 0.119 | RS6 | 0.447 |
| *Acer negundo* | Av. Recoleta | Nov-15 | RH7 | 0.134 | RS7 | 0.420 |
| *Acer negundo* | Av. Recoleta | Nov-15 | RH8 | 0.126 | RS8 | 0.401 |
| *Acer negundo* | Av. Recoleta | Nov-15 | RH9 | 0.126 | RS9 | 0.457 |
| *Acer negundo* | Av. Recoleta | Nov-15 | RH10 | 0.136 | RS10 | 0.479 |
| 1. **Mixed sample group** | | | | | | |
| *Olea europeae* | Recoleta /Zapadores | May-18 | OEH1 Rec-Z | 0.155 | OE-SM/S1 Rec-Z | 0.330 |
| *Olea europeae* | Recoleta/Buenos Aires | May-18 | OEH2 Rec-B | 0.180 | OES2 Rec-B | 0.350 |
| *Brachichyton populneus* | Recoleta/Einstein | May-18 | BrPH1 Rec-E | 0.148 | BrPS1 Rec-E | 0.361 |
| *Quillaja saponaria* | Recoleta/Einstein | May-18 | QSH1 Rec-E | 0.167 | QSS1 Rec-E | 0.375 |
| *Schinus molle* | Recoleta/Zapadores | May-18 | SMH1 Rec-Z | 0.178 |  |  |
| *Cryptocaria alba* | Portugal/Marin | May-18 | CAH1 Por-M | 0.151 | CAS1 Por-M | 0.363 |
| *Acacia caven* | Alameda/Manuel Rodriguez | May-18 | ACH1 Ala-M | 0.147 | ACS1 Ala-M | 0.378 |
| *Maitenus boaria* | Republica/Alameda | May-18 | MBH1 Rep-A | 0.164 | MBS1 Rep-A | 0.358 |
| 1. **Monitoring stations Group** | | | | | | |
| *Quillaja saponaria* | Puente Alto | Nov-16 | MS-PA | 0.161 |  |  |
| *Quillaja saponaria* | El Bosque | Nov-16 | MS-EB | 0.147 |  |  |
| *Quillaja saponaria* | Cerrillos | Nov-16 | MS-C | 0.161 |  |  |
| *Quillaja saponaria* | Parque O'Higgins | Nov-16 | MS-PO | 0.162 |  |  |
| *Quillaja saponaria* | Cerro Navia | Nov-16 | MS-CN | 0.156 |  |  |
| *Quillaja saponaria* | Las Condes | Nov-16 | MS-LC | 0.155 |  |  |
| *Quillaja saponaria* | La Florida | Nov-16 | MS-LF | 0.153 |  |  |
| *Quillaja saponaria* | Independencia | Nov-16 | MS-I | 0.175 |  |  |
| *Quillaja saponaria* | Quilicura | Nov-16 | MS-Q | 0.172 |  |  |
